# Enhancement of Palmarumycins C_12_ and C_13_ Production in Liquid Culture of Endophytic Fungus *Berkleasmium* sp. Dzf12 after Treatments with Metal Ions

**DOI:** 10.3390/ijms14010979

**Published:** 2013-01-07

**Authors:** Yan Mou, Haiyu Luo, Ziling Mao, Tijiang Shan, Weibo Sun, Kaiyi Zhou, Ligang Zhou

**Affiliations:** Department of Plant Pathology, College of Agronomy and Biotechnology, China Agricultural University, Beijing 100193, China; E-Mails: muyan01987@163.com (Y.M.); luohaiyu69@163.com (H.L.); maoziling2011@163.com (Z.M.); shan5400388@163.com (T.S.); sunweibo.1001@163.com (W.S.); cqzhoukaiyi@126.com (K.Z.)

**Keywords:** endophytic fungus, *Berkleasmium* sp. Dzf12, spirobisnaphthalene, palmarumycin C_12_, palmarumycin C_13_, metal ions, calcium ion, copper ion, aluminum ion, center composite design, response surface methodology

## Abstract

The influences of eight metal ions (*i.e*., Na^+^, Ca^2+^, Ag^+^, Co^2+^, Cu^2+^, Al^3+^, Zn^2+^, and Mn^4+^) on mycelia growth and palmarumycins C_12_ and C_13_ production in liquid culture of the endophytic fungus *Berkleasmium* sp. Dzf12 were investigated. Three metal ions, Ca^2+^, Cu^2+^ and Al^3+^ were exhibited as the most effective to enhance mycelia growth and palmarumycin production. When calcium ion (Ca^2+^) was applied to the medium at 10.0 mmol/L on day 3, copper ion (Cu^2+^) to the medium at 1.0 mmol/L on day 3, aluminum ion (Al^3+^) to the medium at 2.0 mmol/L on day 6, the maximal yields of palmarumycins C_12_ plus C_13_ were obtained as 137.57 mg/L, 146.28 mg/L and 156.77 mg/L, which were 3.94-fold, 4.19-fold and 4.49-fold in comparison with that (34.91 mg/L) of the control, respectively. Al^3+^ favored palmarumycin C_12_ production when its concentration was higher than 4 mmol/L. Ca^2+^ had an improving effect on mycelia growth of *Berkleasmium* sp. Dzf12. The combination effects of Ca^2+^, Cu^2+^ and Al^3+^ on palmarumycin C_13_ production were further studied by employing a statistical method based on the central composite design (CCD) and response surface methodology (RSM). By solving the quadratic regression equation between palmarumycin C_13_ and three metal ions, the optimal concentrations of Ca^2+^, Cu^2+^ and Al^3+^ in medium for palmarumycin C_13_ production were determined as 7.58, 1.36 and 2.05 mmol/L, respectively. Under the optimum conditions, the predicted maximum palmarumycin C_13_ yield reached 208.49 mg/L. By optimizing the combination of Ca^2+^, Cu^2+^ and Al^3+^ in medium, palmarumycin C_13_ yield was increased to 203.85 mg/L, which was 6.00-fold in comparison with that (33.98 mg/L) in the original basal medium. The results indicate that appropriate metal ions (*i.e.*, Ca^2+^, Cu^2+^ and Al^3+^) could enhance palmarumycin production. Application of the metal ions should be an effective strategy for palmarumycin production in liquid culture of the endophytic fungus *Berkleasmium* sp. Dzf12.

## 1. Introduction

Plant endophytic fungi colonize interior organs of plants without causing apparent symptoms of pathogenesis [1]. They are responsible for the adaptation of plants to abiotic stresses such as drought, cold, light and metals, as well as to biotic ones such as herbivores, insects and pathogens [2]. Endophytic fungi act as a reservoir of genetic diversity, and have been proven to be a rich source of biologically active natural products [3–6]. They have also been found to produce the same or similar important metabolites produced by the host plants, such as alkaloids, steroids, phenolics, terpenoids and peptides [7].

*Berkleasmium* sp. Dzf12 is an endophytic fungus derived from the rhizomes of *Dioscorea zingiberensis* C. H. Wright (Dioscoreaceae), a well-known traditional Chinese medicinal herb indigenous to the south of China [8,9]. In our previous studies, six spirobisnaphthalenes were obtained from *Berkleasmium* sp. Dzf12, and both palmarumycins C_12_ and C_13_ were found to be the predominant components [8,10]. Palmarumycin C_12_ showed antifungal activity on *Ustilago violacea* and *Eurotium repens* [11]. Palmarumycin C_13_ (also variously named Sch 53514, diepoxin ζ and cladospirone bisepoxide) exhibited obvious antibacterial and antifungal [8,12], antitumor activity, and inhibitory activity on phospholipase D (PLD) [13]. Spirobisnaphthalenes are a rapidly growing group of naphthoquinone derivatives with the interesting structures and various biological activities such as antitumor, antibacterial, antifungal, antileishmanial, enzyme-inhibitory, and other properties to display their potential applications in agriculture, medicine and the food industry [14,15]. These tremendous discoveries about spirobisnaphthalenes attracted the attention of many researchers.

In order to speed up application of palmarumycins C_12_ and C_13_, one of the most important approaches is to increase yields of palmarumycins C_12_ and C_13_ in fermentation culture of *Berkleasmium* sp. Dzf12. Various strategies have been developed to increase metabolite yield in microorganism or plant cultures, which include optimization of medium, utilization of two-phase culture systems, addition of precursors and metal ions, as well as application of elicitation by using polysaccharides and oligosaccharides [16–24]. Many metal ions (*i.e*., K^+^, Na^+^, Mg^2+^, Ca^2+^, Mn^2+^, Fe^2+^, Co^2+^, Ni^+^, Cu^2+^, Zn^2+^ and Mo^+^) are essential for microorganisms. They can interact with microbial cells and may play important functions in cell growth and metabolism [25,26]. The mycelia pellet formation and fumaric acid production were significantly affected by the trace metal ions Mg^2+^, Zn^2+^, Fe^2+^, and Mn^2+^ in fermentation culture of *Rhizopus oryzae* ATCC 20344 [27]. Mycelia growth and polysaccharide production were obviously enhanced by the metal ions Zn^2+^, Se^2+^ and Fe^2+^ in submerged culture of *Ganoderma lucidum* [28].

In our previous studies, obvious enhancement of palmarumycins C_12_ and C_13_ production in the liquid culture of *Berkleasmium* sp. Dzf12 was achieved by using yeast extract and its fractions [29], *in situ* resin adsorption [30], polysaccharides, and oligosaccharides from the host plant *Dioscorea zingiberensis* [10,31]. Spirobisnapthalenes belong to polyketide metabolites produced by some fungi [14]. Biosynthetically, they are generated by the 1,8-dihydroxynaphthalene (DHN) pathway which contains a series of biochemical reactions including oxidation and reduction [32,33]. In this study, we deal with eight metal ions in medium affecting production of palmarumycins C_12_ and C_13_ in liquid culture of *Berkleasmium* sp. Dzf12. Firstly, the single metal ion at its different concentrations was added in medium to screen its enhancing effect. Secondly, three effective metal ions (Ca^2+^, Cu^2+^ and Al^3+^) along with their addition time were studied to obtain the appropriate combination of addition time and concentration for each ion. Lastly, the combination effects of Ca^2+^, Cu^2+^ and Al^3+^ on palmarumycin C_13_ production in liquid culture of *Berkleasmium* sp. Dzf12 were studied by employing statistical method based on the central composite design (CCD) and response surface methodology (RSM) to realize the maximization of palmarumycin C_13_ yield. To the best of our knowledge, the effects of metal ions on palmarumycin production of *Berkleasmium* sp. Dzf12 have not yet been reported. The purpose was to investigate the enhancing effects of the metal ions for palmarumycins C_12_ and C_13_ biosynthesis in liquid culture of *Berkleasmium* sp. Dzf12, as well as to provide data supporting palmarumycin production on a large scale.

## 2. Results and Discussion

### 2.1. Effects of Metal Ions on Mycelia Growth and Palmarumycin Production

Eight metal ions (*i.e*., Na^+^, Ca^2+^, Ag^+^, Co^2+^, Cu^2+^, Al^3+^, Zn^2+^, and Mn^4+^) were added in medium on day 3 of culture. The effects of the metal ions on mycelia growth and palmarumycin production in *Berkleasmium* sp. Dzf12 liquid culture are presented in Table 1. Three metal ions, Ca^2+^, Cu^2+^ and Al^3+^ showed improving effects on the mycelia growth and palmarumycin accumulation. The optimum concentrations to stimulate mycelia growth for Ca^2+^, Cu^2+^ and Al^3+^ were respectively at 20.00, 3.00 and 3.00 mmol/L. However, the optimal concentrations to obviously increase total palmarumycin yield (palmarumycins C_12_ plus C_13_) for Ca^2+^, Cu^2+^ and Al^3+^ were respectively at 10.00, 1.50 and 3.00 mmol/L. Correspondingly, total palmarumycin yields at the optimal concentrations of Ca^2+^, Cu^2+^ and Al^3+^ were respectively at 129.24, 112.26 and 112.82 mg/L, which were 4.00-fold, 3.47-fold, and 3.49-fold of control yield (32.32 mg/L), respectively. Zinc ions (Zn^2+^) slightly stimulated mycelia growth, and had no obvious effect on total palmarumycin production. Among the other metal ions (*i.e*., Na^+^, Ag^+^, Co^2+^ and Mn^4+^), both Ag^+^ and Co^2+^ strongly inhibited mycelia growth, and both Na^+^ and Mn^4+^ slightly inhibited mycelia growth. Correspondingly, total palmarumycin yield of endophyte Dzf12 liquid cultures was decreased after treatment with the metal ions Na^+^, Ag^+^, Co^2+^ and Mn^4+^, respectively. The inhibitory capacity of the metal ions for palmarumycins C_12_ and C_13_ production in Dzf12 liquid culture was in order of Co^2+^ > Ag^+^ > Mn^4+^ > Na^+^. It was concluded that both Co^2+^ and Ag^+^ exhibited the most toxic to *Berkleasmium* sp. Dzf12 by inhibiting mycelia growth and palmarumycins C_12_ and C_13_ production. Three metal ions, Ca^2+^, Cu^2+^ and Al^3+^ were the most effective at enhancing mycelia growth and palmarumycin production. They were selected for further enhancing experiments for palmarumycin production.

### 2.2. Effects of Calcium Ion Addition Time on Mycelia Growth and Palmarumycin Production

**Figure 1:**
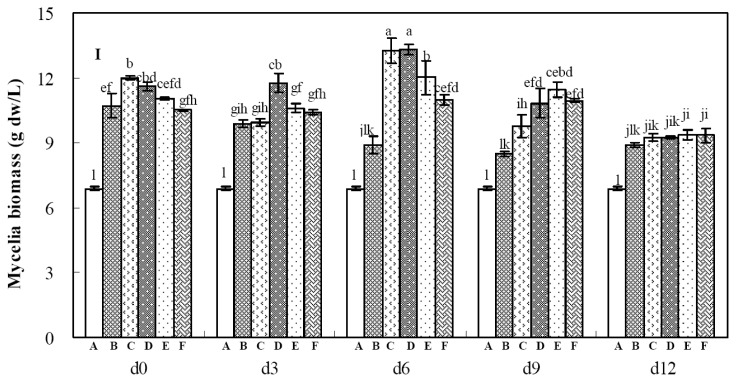

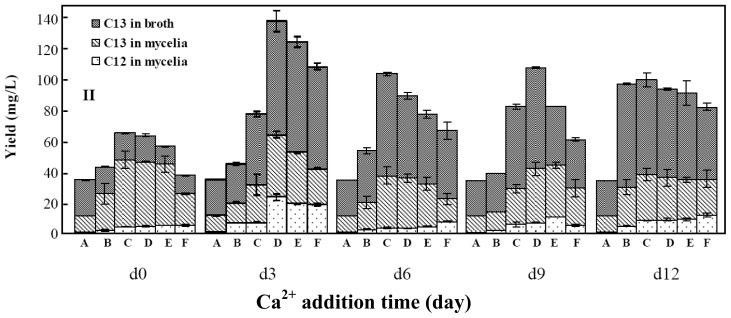
Effects of calcium ion (Ca^2+^) and its addition time on mycelia growth (**I**) and palmarumycins C_12_ and C_13_ production (**II**) in liquid culture of *Berkleasmium* sp. Dzf12. The period of culture lasted for 13 days. The error bars represent standard deviations from three independent samples. Different letters indicate significant differences among the treatments at *p* = 0.05 level. Palmarumycins C_12_ and C_13_ are abbreviated as C_12_ and C_13_, respectively. A, B, C, D, E and F denote 0, 1, 5, 10, 15 and 20 mmol/L of Ca^2+^ concentration in medium, respectively.

Addition time of the metal ions has been considered as a main factor to affect biosynthesis of fungal or plant secondary metabolites [16,18]. Calcium ion (Ca^2+^) was found to be one of the most effective ions to improve mycelia growth and palmarumycin biosynthesis in liquid culture of *Berkleasmium* sp. Dzf12, based on the results shown in Table 1. Hence, Ca^2+^ concentrations in medium and addition time were further optimized. As the three-day-old cultures treated with 10 mmol/L of Ca^2+^ reached an ideal palmarumycin production (129.24 mg/L), the highest concentration of Ca^2+^ in subsequent studies was limited at 20 mmol/L. Figure 1 shows the effects of Ca^2+^ on mycelia growth (Figure 1-I) and palmarumycin yield (Figure 1-II) in *Berkleasmium* sp. Dzf12 liquid cultures, which were dependent on both Ca^2+^ concentrations (1, 5, 10, 15, and 20 mmol/L) and its addition time (added on days 0, 3, 6, 9 and 12). As shown in Figure 1-I, when the cultures were fed with 10 mmol/L of Ca^2+^ on day 6, the mycelia biomass was 1.94-fold of control (13.32 g dw/L *versus* 6.88 g dw/L). Palmarumycin C_13_ was distributed in both mycelia and broth, and palmarumycin C_12_ was only distributed in mycelia. With 10 mmol/L of Ca^2+^ fed on day 3, the highest yields of palmarumycins C_12_ (23.65 mg/L) and C_13_ (113.92 mg/L) were obtained. The total palmarumycin yield (palmarumycins C_12_ plus C_13_) was improved to reach 137.57 mg/L, which was about 3.94-fold of control yield (34.91 mg/L) (Figure 1-II).

Calcium ion (Ca^2+^) plays an essential role in signal transduction as it is one component of the calcium ion channel. Calcium signaling can result in rapid cellular responses, such as cell motility and contractile events, or more slowly paced responses, such as shifts in gene expression and cell division [34]. Ca^2+^ was reported to obviously accelerate the growth of the fungus *Sesarma bidens* [35]. It was also reported to cause thickening of the fungal cell walls of *Botrytis cinerea* [36]. Ca^2+^ uptake is localized in the apex of the tip of fungal hyphae, and Ca^2+^ has been considered to have an important role on fungal tip growth [37–39]. Ca^2+^ was also found to stimulate mycelia growth of *Berkleasmium* sp. Dzf12 in this study. Its action mechanisms to improve mycelia growth and palmarumycin biosynthesis need further investigation.

### 2.3. Effects of Copper Ion Addition Time on Mycelia Growth and Palmarumycin Production

**Figure 2:**
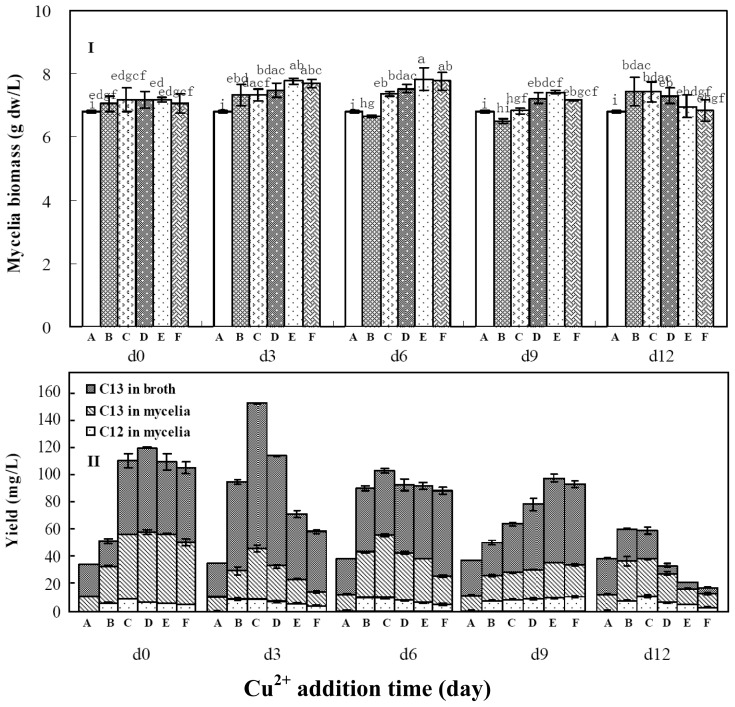
Effects of copper ion (Cu^2+^) and its addition time on mycelia growth (**I**) and palmarumycins C_12_ and C_13_ production (**II**) in liquid culture of *Berkleasmium* sp. Dzf12. The period of culture lasted for 13 days. The error bars represent standard deviations from three independent samples. Different letters indicate significant differences among the treatments at *p* = 0.05 level. Palmarumycins C_12_ and C_13_ are abbreviated as C_12_ and C_13_, respectively. A, B, C, D, E and F denote 0.0, 0.5, 1.0, 1.5, 2.0 and 3.0 mmol/L of Cu^2+^ concentration in medium, respectively.

Copper ion (Cu^2+^) was also found to be one of the most effective ions to improve mycelia growth and palmarumycin biosynthesis in liquid culture of *Berkleasmium* sp. Dzf12 based on the results shown in Table 1. Therefore, Cu^2+^ concentration and addition time were further optimized. As the three-day-old cultures treated with 1.50 mmol/L of Cu^2+^ reached an ideal palmarumycin production (112.26 mg/L), the highest concentration of Cu^2+^ in subsequent studies was limited at 3.00 mmol/L. Figure 2 shows the effects of Cu^2+^ on mycelia growth (Figure 2-I) and palmarumycin yield (Figure 2-II) in *Berkleasmium* sp. Dzf12 liquid cultures, which were dependent on both Cu^2+^ concentrations (0.5, 1.0, 1.5, 2.0, and 3.0 mmol/L) and its addition time (added on days 0, 3, 6, 9 and 12). As shown in Figure 2-I, when the cultures were fed with 0.5–3.0 mmol/L of Cu^2+^ on days 0–12, the mycelia biomass was varied as 6.49–7.84 g dw/L which was higher than that of control (6.88 g dw/L). With 1.0 mmol/L of Cu^2+^ fed on day 3, the highest yields of palmarumycins C_12_ (6.37 mg/L) and C_13_ (139.91 mg/L) were obtained. The total palmarumycin yield (palmarumycins C_12_ plus C_13_) was improved to reach 146.28 mg/L, which was about 4.19-fold of control yield (34.91 mg/L) (Figure 2-II). Cu^2+^ was previously reported to improve the production of laccase by the white-rot fungus *Pleurotus pulmonarius* in solid state fermentation [40].

### 2.4. Effects of Aluminum Ion Addition Time on Mycelia Growth and Palmarymycin Production

**Figure 3:**
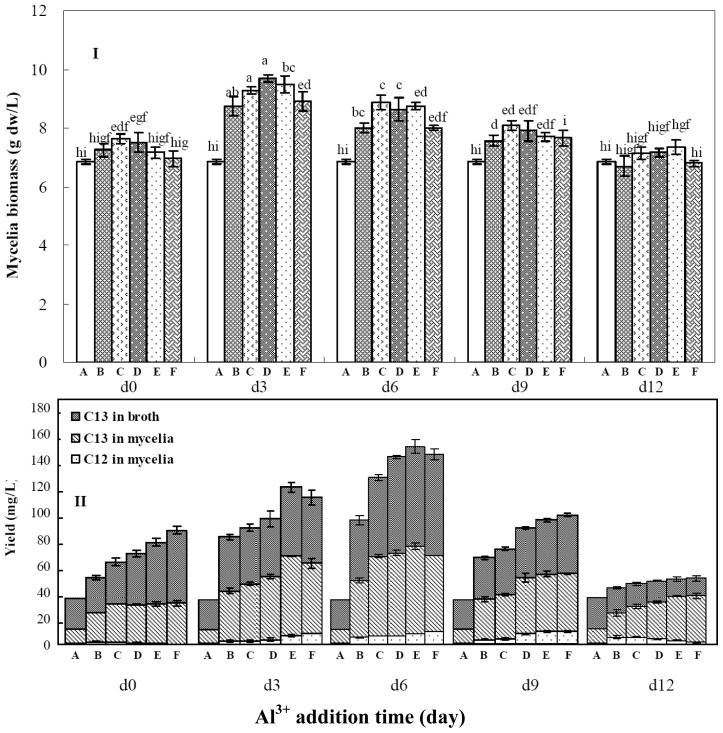
Effects of aluminum ion (Al^3+^) and its addition time on mycelia growth (**I**) and palmarumycins C_12_ and C_13_ production (**II**) in liquid culture of *Berkleasmium* sp. Dzf12. The period of culture lasted for 13 days. The error bars represent standard deviations from three independent samples. Different letters indicate significant differences among the treatments at *p* = 0.05 level. Palmarumycins C_12_ and C_13_ are abbreviated as C_12_ and C_13_, respectively. A, B, C, D, E and F denote 0.0, 0.5, 1.0, 1.5, 2.0 and 2.5 mmol/L of Al^3+^ concentration in medium, respectively.

Effects of aluminum ion (Al^3+^) on mycelia growth and palmarumycin production in liquid culture of *Berkleasmium* sp. Dzf12 are shown in Figure 3. As the three-day-old cultures treated with 1.50 mmol/L of Al^3+^ reached an ideal palmarumycin production (106.21 mg/L), the highest concentration of Al^3+^ in subsequent studies was limited at 2.50 mmol/L. Figure 3 shows the effects of Cu^2+^ on mycelia growth (Figure 3-I) and palmarumycin yield (Figure 3-II) in *Berkleasmium* sp. Dzf12 liquid cultures, which were dependent on both Al^3+^ concentrations (0.5, 1.0, 1.5, 2.0, and 2.5 mmol/L) and its addition time (added on days 0, 3, 6, 9 and 12). As shown in Figure 3-I, when the cultures were fed with 1.5 mmol/L of Al^3+^ on day 3, the mycelia biomass was 9.69 g dw/L which was higher than that of control (6.88 g dw/L). With 2.0 mmol/L of Al^3+^ fed on day 6, the highest yields of palmarumycins C_12_ (8.28 mg/L) and C_13_ (148.49 mg/L) were obtained. The total palmarumycin yield (palmarumycins C_12_ plus C_13_) was improved to reach 156.77 mg/L, which was about 4.49-fold of control yield (34.91 mg/L) (Figure 3-II).

Palmarumycin C_13_ production was mainly enhanced when Al^3+^ concentration was lower than 4 mmol/L (Table 2 and Figure 3). When Al^3+^ concentration was higher than 4 mmol/L, palmarumycin C_12_ production was favored. The ratio of palmarumycin C_12_ yield to palmarumycin C_13_ yield was gradually increased (Table 2). Biosynthetically, palmarumycin C_12_ was considered as the precursor of palmarumycin C_13_ [32,33]. Palmarumycin C_12_ was converted to palmarumycin C_13_ by oxidation reaction in mycelia cells. It is possible that the enzyme activity for oxidation was suppressed as the aluminum ion concentration was higher than 4 mmol/L. While aluminum ion concentration was higher than 8 mmol/L, the total yield of palmarumyicns C_12_ plus C_13_ gradually decreased. It is possible that high Al^3+^ concentration leads to the suppression of other enzymatic activities in the biosynthetic pathway of palmarumycins besides the oxidation enzyme. The results should be beneficial for us to prepare palmarumycin C_12_ or palmarumycin C_13_ by regulating activity of the oxidation enzyme with aluminum ions. Al^3+^ at a high concentration is usually toxic to plants, animals and microorganisms [35]. For example, Al^3+^ completely inhibited the growth of the fungus *Sesarma bidens* at 100 mg/L AlCl_3_ in medium [41]. However, it possesses a variety of biological activities at low concentrations [42]. Al^3+^ enhanced the conversion of (β-isoxazolin-5-on-2-yl)-alanine (BIA) into β-*N*-oxalyl-L-α,β-diaminopropionic acid (β-ODAP) in the callus tissues derived from leaf explants of *Lathyrus sativus* [43]. Production of some plant secondary metabolites can be elicited by Al^3+^. For example, resveratrol biosynthesis in grapevine (*Vitis vinifera*) was stimulated by Al^3+^ at a concentration of 0.05% AlCl_3_ in medium [44]. Tropane alkaloid production and antioxidant system activity in micropropagated *Datura innoxia* plantlets were enhanced by Al^3+^ as an abiotic elicitor [45]. It has not yet been reported how aluminum ions suppress the enzyme-based oxidation. Aluminum ions directly joining the biotransformation step from palmarumycin C_12_ to palmarumycin C_13_ needs further clarification.

### 2.5. Combination Effects of Calcium, Copper and Aluminum Ions on Palmarumycin C_13_ Production

According to the above single-factor experiments, Ca^2+^, Cu^2+^ and Al^3+^ all showed their obvious effects on palmarumycin C_13_ production in liquid culture of *Berkleasmium* sp. Dzf12, and palmarumycin C_13_ yield was in the majority of palmarumycins C_12_ plus C_13_ yield (Figures 1-II, 2-II and 3-II). So the suitable concentrations of Ca^2+^, Cu^2+^ and Al^3+^ in medium for palmarumycin C_13_ production were further determined by central composite design (CCD) experiments and response surface methodology (RSM). Five levels of each variable were set by the software of Design Expert, which are presented in Table 3. Subsequently, 20 trials of CCD were carried out to optimize palmarumycin C_13_ yield. The results of CCD experiments were summarized in Table 4. Palmarumycin C_13_ yield displayed considerable variations from 163.22 to 209.64 mg/L, respectively, depending upon the changes of variables. Based on the results of CCD experiments, a second-order polynomial regression model between palmarumycin C_13_ yield and the test independent variables by software of Design Expert was shown in Equation 1.

(1) $$\begin{array}{c} Y=206.31-8.70x_{1}-0.06x_{2}+1.58x_{3}+5.39x_{1}x_{2}-1.02x_{1}x_{3}+\\ 1.63x_{2}x_{3}-10.68{x_{1}}^{2}-4.42{x_{2}}^{2}-8.53{x_{3}}^{2} \end{array}$$

In Equation 1,*Y* represented palmarumycin C_13_ yield (mg/L), and *x*_1_, *x*_2_ and *x*_3_ were the coded values of the test variables, the concentrations (mmol/L) of Ca^2+^, Cu^2+^ and Al^3+^, respectively.

In order to determine whether the quadratic regression model was significant or not, the ANOVA analysis was conducted, which was summarized in Table 5. The ANOVA of the quadratic regression model demonstrated that the model was highly significant, evident from Fisher’s test with a very high model *F*-value (84.65) but a very low *p*-value (*p* < 0.0001). The goodness of the model was examined by the determination coefficients (*R*^2^) and multiple correlation coefficients (*R*). For palmarumycin C_13_ yield, the value (0.9754) of the determination coefficient adj-*R*^2^ demonstrated that total variation of 97.54% was attributed to the test independent variables and only about 2.46% of the total variation could not be explained by the model. The value of *R* was closer to 1, the fitness of the model was better [46]. In this study, the multiple correlation coefficient (adj-*R*) of the model was 0.9876, indicating a good agreement between the experimental and predicted values. The lack-of-fit measured the failure of the model to represent the data in the experimental domain at points which were not included in the regression [47]. The *F*-value for lack-of-fit was 0.86 and the corresponding *p*-value was 0.56 (> 0.05), which implied each lack-of-fit was not significant relative to the pure error due to noise. Insignificant lack-of-fit confirmed the validity of the model.

The coefficients of the quadratic polynomial model, along with their corresponding *p*-values, are calculated and presented in Table 6. The *p*-value was used as a tool to check the significance of each coefficient, which also indicated the interaction strength between each independent parameter [48]. If the *p*-value was smaller, the significance of the corresponding coefficient should be bigger. It can be seen from Table 6 that most of regression coefficients of the quadratic polynomial models were significant with low *p*-values.

**Figure 4:**
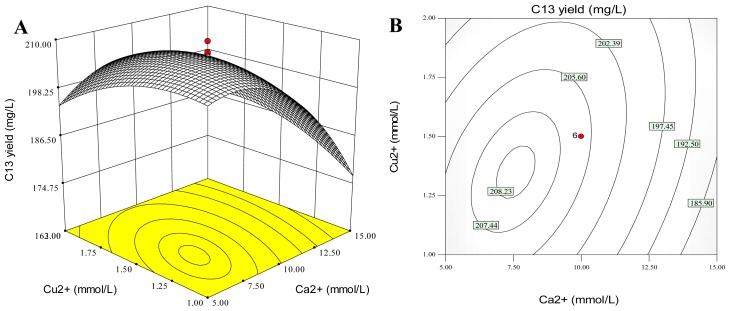

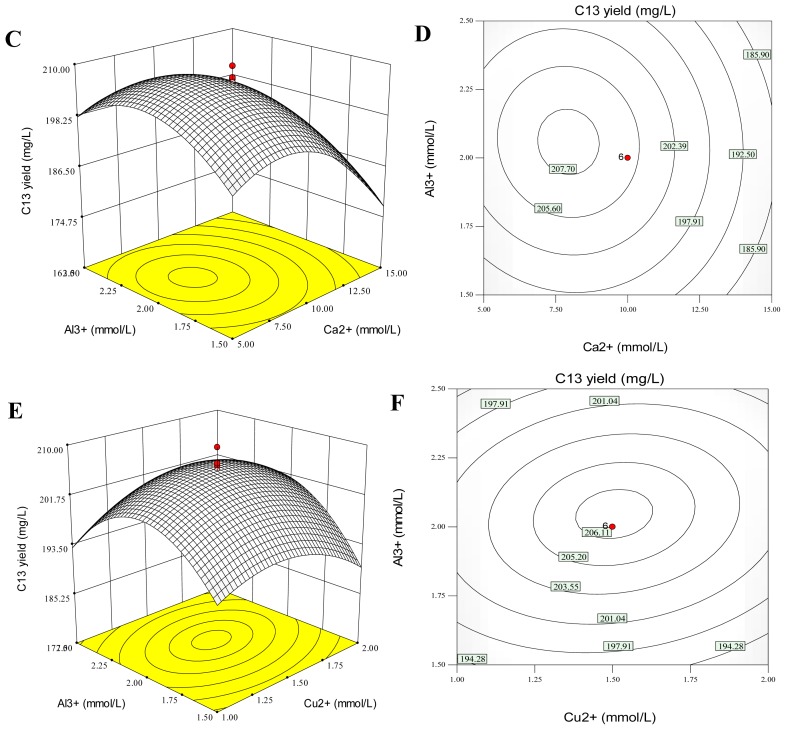
Three-dimensional response surfaces (**A**, **C** and **E**) and two-dimensional contour plots (**B**, **D** and **F**) of palmarumycin C_13_ yield (mg/L) *versus* the test variables (mmol/L): Ca^2+^ and Cu^2+^ (**A** and **B**); Ca^2+^ and Al^3+^ (**C** and **D**); Cu^2+^ and Al^3+^ (**E** and **F**). Palmarumycin C_13_ was abbreviated as C_13_.

The three-dimensional (3D) response surface and two-dimensional (2D) contour plots are the graphical representations of the quadratic polynomial regression equation [49]. They provide a method to visualize the relationship between the responses and the experimental levels of each variable, and the interactions between any two test variables from the circular or elliptical nature of contour [50]. A circular contour plot indicates that the interactions between the corresponding variables are negligible. An elliptical nature of the contour plots indicates that the interactions between the corresponding variables are significant [51]. In this study, the 3D response surfaces and 2D contour plots of palmarumycin C_13_ field (mg/L) *versus* the metal ion concentrations (mmol/L) are presented in Figure 4, which were generated by employing the software of Design-Expert. Analyses of the 3D response surfaces and their corresponding 2D contour plots allowed us to conveniently investigate the interactions between any two variables, and locate the optimum ranges of the variables efficiently such that the response was maximized. The maximum predicted response was indicated by the surface confined in the smallest ellipse in the contour diagram.

Figure 4A,B graphed the effects of Ca^2+^ and Cu^2+^ on palmarumycin C_13_ yield and their interactions when Al^3+^ concentration was fixed at zero level (2.0 mmol/L). A full elliptic contour in Figure 4B was observed, indicating a significant interaction between Ca^2+^ and Cu^2+^ for palmarumycin C_13_ production. When the concentrations of Ca^2+^ and Cu^2+^ in medium were increased from the lowest levels to the highest levels, palmarumycin C_13_ yield was increased initially and then decreased. Similar results appeared in Figure 4C,D, as well as in Figure 4E,F. Palmarumycin C_13_ yield was initially augmented and then decreased when the concentrations of two metal ions (either Ca^2+^ and Al^3+^ or Cu^2+^ and Al^3+^) varied from the lowest levels to the highest levels. The interactions either between Ca^2+^ and Al^3+^ or between Cu^2+^ and Al^3+^ were not significant. They were consistent with the analyses of coefficients of the regression equation (Table 6).

By analyzing the 3D response surface and 2D contour plots, the corresponding point to the maximum of palmarumycin C_13_ yield should locate on the peak of the response surface, which projected in the smallest ellipse in the contour diagram [52]. Hence, the optimal ranges of the concentrations of Ca^2+^, Cu^2+^ and Al^3+^ in medium for realizing the maximization of palmarumycin C_13_ yield were calculated by the software Design Expert as follows: 5.94 to 9.06 mmol/L for Ca^2+^, 1.24 to 1.63 mmol/L for Cu^2+^, and 1.86 to 2.15 mmol/L for Al^3+^.

By solving the inverse matrix of the regression polynomial equation (Equation 1) employing the software of Design-Expert, the optimum values for palmarumycin C_13_ yield of the test parameters in uncoded units were obtained as follows: Ca^2+^ as 7.58 mmol/L, Cu^2+^ as 1.36 mmol/L, and Al^3+^ as 2.05 mmol/L. Under the optimum conditions, the predicted palmarumycin C_13_ yield reached the maximum (208.49 mg/L). Under the determined conditions, a mean value of palmarumycin C_13_ yield of 203.85 mg/L (*n* = 5) was obtained from the actual experiments, slightly lower than the predicted maximum value (208.49 mg/L), and about 6.00-fold of the original yield (33.98 mg/L) in the original basal medium. Based on the Student *t*-test, the above model was satisfactory and adequate for reflecting the expected optimization as no significant difference was observed between the predicted maximum palmarumycin C_13_ yield and the experimental one.

## 3. Experimental Section

### 3.1. Endophytic Fungus and Culture Conditions

The endophytic fungus *Berkleasmium* sp. Dzf12 (GenBank accession number EU543255) was isolated from the healthy rhizomes of the medicinal plant *Dioscorea zingiberensis* C. H. Wright (Dioscoreaceae) in our previous study [8,9]. The living culture has been deposited in China General Microbiological Culture Collection Center (CGMCC) under the number of CGMCC 2476. It was also maintained on potato dextrose agar (PDA) slants at 4 °C. For preparation of the inoculum, four disks (about 5 mm) of the mycelia of Dzf12 were transferred into each Erlenmeyer flask (300 mL) containing 100 mL of potato dextrose broth. After 4 days’ cultivation on a rotary shaker in darkness at 150 rpm and 25 °C, the seed suspension cultures in three percent (*v*/*v*) were inoculated in Erlenmeyer flasks (150 mL) containing 30 mL of fermentation medium, which composed of glucose 40 g/L, peptone 10 g/L, KH_2_PO_4_ 1.0 g/L, MgSO_4_·7H_2_O 0.5 g/L and FeSO_4_·7H_2_O 0.05 g/L, pH 6.5. The Erlenmeyer flasks were incubated on a rotary shaker in darkness at 150 rpm and 25 °C for 13 days.

### 3.2. Application of the Metal Ions

Stock metal ion solutions were prepared by dissolving each inorganic salt (*i.e*., NaCl, CaCl_2_·2H_2_O, AgNO_3_, CoCl_2_·6H_2_O, CuCl_2_·2H_2_O, AlCl_3_·6H_2_O, ZnCl_2_, and MnCl_4_·4H_2_O) in distilled water. The solutions were sterilized by filtrating through a microfilter (pore size, 0.22 μm), diluted with sterile water into different concentrations, and then stored at 4 °C before use. Based on our preliminary experiments (data not shown), the metal ion solutions were added to three-day-old cultures. 100 μL of the stock solution was used as inoculum in 30 mL medium in the experiments with varying amounts of NaCl (final concentrations of Na^+^ in medium as 5, 10, 20 mmol/L), CaCl_2_·2H_2_O (final concentrations of Ca^2+^ in medium as 5, 10, 20 mmol/L), AgNO_3_ (final concentrations of Ag^+^ in medium as 0.05, 0.10 and 0.50 mmol/L), CoCl_2_·6H_2_O (final concentrations of Co^2+^ in medium as 0.05, 0.10 and 0.50 mmol/L), CuCl_2_·2H_2_O (final concentrations of Cu^2+^ in medium as 0.50, 1.50 and 3.00 mmol/L), AlCl_3_·6H_2_O (final concentrations of Al^3+^ in medium as 0.50, 1.50 and 3.00 mmol/L), ZnCl_2_ (final concentrations of Zn^2+^ in medium as 2.00, 4.00 and 8.00 mmol/L), MnCl_4_·4H_2_O (final concentrations of Mn^4+^ in medium as 2.00, 4.00 and 8.00 mmol/L), respectively. As Ca^2+^, Cu^2+^ and Al^3+^ were found to be the most effective metal ions (data shown in Table 1), they were applied in the next experiments at five concentrations (1, 5, 10, 15 and 20 mmol/L for Ca^2+^; 0.5, 1.0, 1.5, 2.0 and 3.0 mmol/L for Cu^2+^; 0.5, 1.0, 1.5, 2.0 and 2.5 mmol/L for Al^3+^) on days 0, 3, 6, 9 and 12 of culture, respectively. Furthermore, Al^3+^ was added on day 3 of culture at six concentrations (2, 4, 6, 8, 10 and 12 mmol/L) to investigate its effect on palmarumycins C_12_ and C_13_ production. For investigating the combination effects of Ca^2+^, Cu^2+^ and Al^3+^ on palmarumycin C_13_ production, the final concentrations of the metal ions were set by the software of Design Expert and presented in Table 3.

### 3.3. Determination of Mycelia Biomass

The mycelia of *Berkleasmium* sp. Dzf12 were separated from the liquid medium by filtration under vacuum and rinsed thoroughly with distilled water, and then dried at 50–55 °C in an oven to a constant dry weight (dw).

### 3.4. Extraction and Quantification of Palmarumycins C_12_ and C_13_

Palmarumycin extraction and determination were carried out as previously described [10,29–31]. Briefly, 50 mg of dry mycelia powder was added into a tube with 5 mL of methanol-chloroform (9:1, *v*/*v*), and then subjected to ultrasonic treatment (three times, 60 min each). After removal of the solid by filtration, the filtrate was evaporated to dryness and re-dissolved in 1 mL of methanol. For quantitative analysis of palmarumycins C_12_ and C_13_ in broth, 3 mL of the culture broth without mycelia was evaporated to dryness and extracted with 5 mL of methanol-chloroform (9:1, *v*/*v*) in an ultrasonic bath (three times, 60 min each), and the liquid extract was then evaporated to dryness and re-dissolved in 1 mL of methanol.

Palmarumycin content was analyzed by the high performance liquid chromatography (HPLC) system (Shimadzu, Japan), which consisted of two LC-20AT solvent delivery units, an SIL-20A autosampler, an SPD-M20A photodiode array detector, and CBM-20Alite system controller. The reversed-phase Agilent TC-C_18_ column (250 mm × 4.6 mm i.d., particle size 5 μm) was used for separation by using a mobile phase of methanol-H_2_O (50:50, *v*/*v*) at a flow rate of 1.0 mL/min. The temperature was maintained at 40 °C, and UV detection at 226 nm. The sample injection volume was 10 μL. The LCsolution multi-PDA workstation was employed to acquire and process chromatographic data. Palmarumycins C_12_ and C_13_ were detected and quantified with the standards prepared according to the method of Cai *et al.* [8] and Li *et al.* [10].

### 3.5. Experimental Design for the Combination Effects of the Metal Ions

Central composite design (CCD) experiments and response surface methodology (RSM) were employed to optimize the concentrations of Ca^2+^, Cu^2+^ and Al^3+^ in the fermentation medium for realizing the maximization of palmarumycin C_13_ yield by the software of Design-Expert. The metal ion solutions were added to 3-day-old cultures. 100 μL of the stock solution was used as inoculum in 30 mL medium in the experiments with varying amounts of CaCl_2_·2H_2_O, CuCl_2_·2H_2_O, and AlCl_3_·6H_2_O, respectively. Each independent variable (final metal ion concentration) in the CCD experiments was studied at five coded levels (−1.682, −1, 0, +1, +1.682), which is represented in Table 3. The independent variable was expressed as *X*_i_, which was coded as *x_i_*, according to the following equation (Equation 2):

(2) $$x_{i}=(X_{i}-X_{0})/\Delta X,i=1,2,3$$

where *x_i_* is the coded value of the variable *X_i_*, while *X*_0_ is the value of *X_i_* at the center point, and Δ*X* is the step change of an independent variable.

CCD in this experimental design consisted of 20 trials which were carried out in a random order in triplicate that was necessary to estimate the variability of measurements, which are presented in Table 4. Five replicates at the center point of the design were carried out to allow for estimation of a pure error sum of squares. Palmarumycin C_13_ yield was recorded as the mean of triplicates, which was taken as the response value.

Based on the CCD experimental data, a second-order polynomial model was established, which correlated the relationship between palmarumycin C_13_ and the test independent variables. The relationship could be expressed by the following equation (Equation 3):

(3) $$Y=a_{0}+a_{1}x_{1}+a_{2}x_{2}+a_{3}x_{3}+a_{12}x_{1}x_{2}+a_{13}x_{1}x_{3}+a_{23}x_{2}x_{3}+a_{11}{x_{1}}^{2}+a_{22}{x_{2}}^{2}+a_{33}{x_{3}}^{2}$$

where *Y* is the predicted response value; *a*_0_ is the intercept term; *x*_1_, *x*_2_ and *x*_3_ are test independent variables; *a*_1_, *a*_2_ and *a*_3_ are linear coefficients; *a*_12_, *a*_13_ and *a*_23_ are cross-product coefficients; and *a*_11_, *a*_22_ and *a*_33_ are the quadratic term coefficients. All of the coefficients of the second polynomial model and the responses obtained from the experimental design were subjected to multiple nonlinear regression analyses.

The fitness of the second-order polynomial model equation was evaluated by the coefficient (*R*^2^) of determination. The analysis of variance (ANOVA) and test of significance for regression coefficients were conducted by *F*-test. In order to visualize the relationship between the response values and test independent variables, the fitted polynomial equation was separately expressed as 3D response surfaces and 2D contour plots by the software of Design Expert [53,54].

### 3.6. Statistical Analysis

All experiments were carried out in triplicate, and the results were represented by their mean values and the standard deviations (SD). The data were submitted to analysis of variance (one-way ANOVA) to detect significant differences by PROC ANOVA of SAS version 8.2. The term significant has been used to denote the differences for which *p* ≤ 0.05.

## 4. Conclusions

In this work, three metal ions (Ca^2+^, Cu^2+^ and Al^3+^) were screened from eight metal ions to show their stimulatory effects on mycelia growth and palmarumycins C_12_ and C_13_ production in liquid culture of the endophytic fungus *Berkleasmium* sp. Dzf12. When Ca^2+^ was applied to the medium at 10.0 mmol/L on day 3, Cu^2+^ to the medium at 1.0 mmol/L on day 3, Al^3+^ to the medium at 2.0 mmol/L on day 6, the maximal yields of palmarumycins C_12_ plus C_13_ were obtained as 137.57 mg/L, 146.28 mg/L and 156.77 mg/L, which were 3.94-fold, 4.19-fold and 4.49-fold in comparison with that (34.91 mg/L) of control, respectively. Meanwhile, Al^3+^ favored palmarumycin C_12_ production when its concentration was higher than 4 mmol/L. Ca^2+^ had an enhancing effect on mycelia growth of *Berkleasmium* sp. Dzf12. This is the first time the enhancing effect of Ca^2+^, Cu^2+^ and Al^3+^ on secondary metabolite production of fungi has been reported. Zn^2+^ slightly stimulated mycelia growth, and had no obvious effect on total palmarumycin production. The mycelia growth was strongly inhibited by Ag^+^ and Co^2+^, and slightly inhibited by Na^+^ and Mn^4+^. The inhibitory capacity of the metal ions Na^+^, Ag^+^, Co^2+^ and Mn^4+^ for palmarumycins C_12_ and C_13_ production was in the order of Co^2+^ > Ag^+^ > Mn^4+^ > Na^+^. Based on the results in this study, it was concluded that an enhancing effect could be determined by the metal ions (*i.e.*, Ca^2+^, Cu^2+^ and Al^3+^) along with the combination of their addition time and concentration. The combination of Ca^2+^, Cu^2+^ and Al^3+^ for palmarumycin C_13_ production in liquid culture of *Berkleasmium* sp. Dzf12 was further optimized by employing a statistical method based on CCD and RSM. By solving the quadratic regression equation between palmarumycin C_13_ yield and three variables (*i.e*., the concentrations of Ca^2+^, Cu^2+^ and Al^3+^), the optimal concentrations of Ca^2+^, Cu^2+^ and Al^3+^ in medium for palmarumycin C_13_ production were determined as 7.58, 1.36 and 2.05 mmol/L, respectively. Under the optimum conditions, the predicted maximum palmarumycin C_13_ yield reached 208.49 mg/L. The predicted maximum values for palmarumycin C_13_ yield showed no significant differences from the experimental ones. By optimizing the combination of three metal ions in medium, palmarumycin C_13_ yield was increased to 203.85 mg/L, which was 6.00-fold in comparison with that (33.98 mg/L) in the original basal medium. The results indicate that enhancement of palmarumycin accumulation in liquid culture of *Berkleasmium* sp. Dzf12 by the metal ions should be an effective strategy for large-scale production of palmarumycins in the future. As the combination effects of only three metal ions (*i.e*., Ca^2+^, Cu^2+^ and Al^3+^) have been studied for their enhancing effects on palmarumycin C_13_ production in this work, more components in the medium, as well as other parameters like pH, temperature, oxygen supply, and other ionic strengths, should be considered in future work. Furthermore, the action mechanisms of the metal ions on palmarumycin biosynthesis also need to be studied in detail. After a series of optimizations for palmarumyicn biosynthesis conditions, we could achieve the final optimized medium for maximum production of palmarumycins by natural fermentation of the endophytic fungus *Berkleasmium* sp. Dzf12. Furthermore, some biosynthetic mechanisms of palmarumycins should be helpful for our chemical synthetic methods, though some of those achieved are, at present, neither economically viable nor environmentally friendly [14,15].

## Figures and Tables

**Table 1:** Effects of eight metal ions on mycelia growth and palmarumycin production in liquid culture of *Berkleasmium* sp. Dzf12. Each metal ion was added in medium on day 3 of culture. The period of culture lasted for 13 days. Palmarumycins C_12_ and C_13_ are abbreviated as C_12_ and C_13_, respectively.

Metal ion	Conc. (mmol/L)	Mycelia biomass (g dw/L)	C_12_ yield (mg/L)	C_13_ yield (mg/L)	C_12_ plus C_13_ yield (mg/L)
CK	0.00	6.79 ± 0.08 cde	1.29 ± 0.03 fgh	31.03 ± 0.06 h	32.32 ± 0.13 g

Na^+^	5.00	5.81 ± 0.18 fg	0.66 ± 0.01 ghi	16.10 ± 0.21 ij	16.76 ± 0.32 ij
	10.00	5.83 ± 0.13 fg	3.00 ± 0.17 e	19.25 ± 0.34 i	22.25 ± 1.51 hi
	20.00	5.96 ± 0.25 efg	2.06 ± 0.19 e	19.39 ± 0.15 i	21.45 ± 0.38 hi

Ca^2+^	5.00	7.26 ± 0.17 bcd	4.98 ± 0.21 d	91.57 ± 1.76 d	96.55 ± 2.66 c
	10.00	7.32 ± 0.44 bcd	14.76 ± 0.31 a	114.48 ± 6.76 a	129.24 ± 7.12 a
	20.00	7.58 ± 0.10 bc	13.79 ± 0.90 a	83.48 ± 2.37 e	97.27 ± 3.63 c

Ag^+^	0.05	3.21 ± 0.29 k	0.00 ± 0.00 i	0.67 ± 0.13	l 0.67 ± 0.13 k
	0.10	3.17 ± 0.26 k	0.00 ± 0.00 i	0.44 ± 0.16 l	0.44 ± 0.16 k
	0.50	2.99 ± 0.31 k	0.00 ± 0.00 i	0.32 ± 0.09 l	0.32 ± 0.09 k

Co^2+^	0.05	4.35 ± 0.50 ij	0.00 ± 0.00 i	0.30 ± 0.02 l	0.30 ± 0.02 k
	0.10	4.29 ± 0.18 ij	0.00 ± 0.00 i	0.39 ± 0.09 l	0.39 ± 0.09 k
	0.50	3.52 ± 0.76 jk	0.00 ± 0.00 i	0.21 ± 0.10 l	0.21 ± 0.10 k

Cu^2+^	0.50	7.51 ± 0.33 b	1.42 ± 0.18 fg	78.97 ± 0.26 e	80.39 ± 4.13 d
	1.50	7.67 ± 0.22 b	6.65 ± 0.06 c	105.61 ± 1.70 b	112.26 ± 3.07 b
	3.00	7.82 ± 0.13 b	4.62 ± 0.21d	63.84 ± 0.86 f	68.46 ± 1.06 e

Al^3+^	0.50	7.08 ± 0.33 bcd	2.20 ± 0.86 ef	51.49 ± 2.16 g	53.69 ± 4.77 f
	1.50	8.09 ± 0.29 ab	8.38 ± 0.85 b	97.83 ± 3.67 cd	106.21 ± 5.50 bc
	3.00	8.82 ± 0.33 a	9.00 ± 0.62 d	103.82 ± 5.80 bc	112.82 ± 9.41 b

Zn^2+^	2.00	7.09 ± 0.27 bcd	0.32 ± 0.15 ghi	15.85 ± 1.97 ij	16.17 ± 1.57 ij
	4.00	7.66 ± 0.18 bc	0.63 ± 0.13 ghi	31.42 ± 2.54 h	32.05 ± 1.63 g
	8.00	6.49 ± 0.39 def	0.59 ± 0.14 ghi	29.67 ± 1.58 h	30.26 ± 2.01 gh

Mn^4+^	2.00	4.79 ± 0.28 hi	0.14 ± 0.01 hi	7.23 ± 0.42 kl	7.37 ± 0.77 jk
	4.00	5.43 ± 0.51 gh	0.15 ± 0.02 hi	7.43 ± 0.81 kl	7.58 ± 0.52 jk
	8.00	5.82 ± 0.49 fg	0.20 ± 0.00 hi	10.10 ± 0.19 kj	10.30 ± 1.01 jk

Note: CK, a control without addition of the test metal ions; C_12_, palmarumycin C_12_; C_13_, palmarumycin C_13_; the values are expressed as means ± standard deviations (*n* = 3). Different letters indicate significant differences among the treatments in each column including different metal ions and their concentrations at *p* = 0.05 level.

**Table 2:** Effects of aluminum ion on mycelia growth and palmarumycins C_12_ and C_13_ production in liquid culture of *Berkleasmium* sp. Dzf12. Aluminum ion was added in medium on day 3 of culture. The period of culture lasted for 13 days. Palmarumycins C_12_ and C_13_ are abbreviated as C_12_ and C_13_, respectively.

Aluminum ion conc. (mmol/L)	Mycelia biomass (g dw/L)	C_12_ yield (mg/L)	C_13_ yield (mg/L)	C_12_ plus C_13_ yield (mg/L)	Ratio of C_12_ yield to C_13_ yield
0	7.35 ± 0.17 d	1.83 ± 0.48 c	38.14 ± 3.21 d	39.97 ± 3.07 d	0.05
2	11.16 ± 0.24 abc	9.98 ± 1.47 c	117.26 ± 5.70 b	127.24 ± 4.25 c	0.09
4	11.85 ± 0.41 ab	14.94 ± 1.25 c	166.27 ± 3.42 a	181.21 ± 2.87 b	0.09
6	11.76 ± 1.21 ab	106.59 ± 5.31 b	162.93 ± 4.08 a	269.52 ± 5.19 a	0.65
8	13.17 ± 0.39 a	224.33 ±10.08 a	59.23 ± 2.72 c	283.56 ± 8.08 a	3.79
10	10.51 ± 1.01 bc	118.85 ± 3.93 b	4.19 ± 0.93 e	123.04 ± 3.91 c	28.36
12	9.49 ± 0.98 cd	115.55 ± 2.97 b	3.58 ± 0.13 e	119.13 ± 2.88 c	32.28

Note: C_12_, palmarumycin C_12_; C_13_, palmarumycin C_13_; the values are expressed as means ± standard deviations (*n* = 3). Different letters indicate significant differences among the treatments in each column at *p* = 0.05 level.

**Table 3:** Coded values (*x*) and uncoded values (*X*) of variables in the central composite design (CCD) experiments.

Variable (mmol/L)	Symbol		Coded level				
	
	Uncoded	Coded	−1.682	−1	0	1	+1.682
Ca^2+^	*X*_1_	*x*_1_	1.59	5	10	15	18.41
Cu^2+^	*X*_2_	*x*_2_	0.65	1.0	1.5	2.0	2.34
Al^3+^	*X*_3_	*x*_3_	1.16	1.5	2.0	2.5	2.84

**Table 4:** CCD experimental matrix and the response values for the experiments.

Run	*x*_1_	*x*_2_	*x*_3_	Palmarumycin C_13_ yield (mg/L)		

				Experimental *Y*_e_	Predicted *Y*_p_	*Y*_e_ − *Y*_p_
1	1.68	0	0	163.22	161.47	1.75
2	0	0	−1.68	177.98	179.53	−1.55
3	0	0	0	202.40	206.31	−3.91
4	−1	1	1	189.29	190.14	−0.85
5	0	0	0	206.74	206.31	0.43
6	1	−1	1	166.33	167.58	−1.25
7	−1	1	−1	182.75	181.70	1.05
8	0	0	0	209.64	206.31	3.33
9	1	1	1	179.77	181.50	−1.73
10	0	0	0	205.98	206.31	−0.33
11	0	0	0	207.10	206.31	0.79
12	0	1.68	0	194.98	193.70	1.28
13	0	0	1.68	186.67	184.83	1.84
14	1	−1	−1	170.37	169.72	0.65
15	−1.68	0	0	189.26	190.72	−1.46
16	0	−1.68	0	192.91	193.90	−0.99
17	−1	−1	−1	197.38	195.86	1.52
18	−1	−1	1	198.36	197.78	0.58
19	1	1	−1	176.34	177.12	−0.78
20	0	0	0	205.94	206.31	−0.37

**Table 5:** Palmarumycin C_13_ yield analysis of variance (ANOVA) for the fitted quadratic polynomial model.

Source	Sum of squares	d.f.	Mean square	*F* value	Probability *p* >	*F*
Model	3890.94	9	432.33	84.65	<0.0001	
Residual	51.08	10	5.12			
Lack of fit	23.61	5	4.72	0.86	0.56	
Pure error	27.46	5	5.49			
Corrected total	3942.02	19				

*R*^2^ = 0.9870; adj-*R*^2^ = 0.9754; *R* = 0.9935; adj-*R* = 0.9876; CV (%) = 1.19.

**Table 6:** Regression coefficient and their significance test of the quadratic polynomial model for palmarumycin C_13_ yield.

Model Term	Coefficient estimate	Standard error	Sum of Squares	d.f.	Mean square	*F* value	Probability *p* > *F*
Intercept	206.31	0.92					
*x*_1_	−8.70	0.61	1032.61	1	1032.61	202.17	<0.0001
*x*_2_	−0.060	0.61	0.049	1	0.049	0.0095	0.9243
*x*_3_	1.58	0.61	33.92	1	33.92	6.64	0.0275
*x*_1_*x*_2_	5.39	0.80	232.41	1	232.41	45.50	<0.0001
*x*_1_*x*_3_	−1.017	0.80	8.27	1	8.27	1.62	0.2320
*x*_2_*x*_3_	1.63	0.80	21.23	1	21.23	4.16	0.0688
*x*_1_^2^	−10.68	0.60	1644.34	1	1644.34	321.94	<0.0001
*x*_2_^2^	−4.42	0.60	281.79	1	281.79	55.17	<0.0001
*x*_3_^2^	−8.53	0.60	1048.77	1	1048.77	205.34	<0.0001
